# Depression and Anxiety as Comorbidities in Chronic Obstructive Pulmonary Disease: A Comprehensive Narrative Review

**DOI:** 10.3390/healthcare13182344

**Published:** 2025-09-18

**Authors:** Iulian-Laurențiu Buican, Alina-Cătălina Buican-Chirea, Mădălina Iuliana Mușat, Costin Teodor Streba

**Affiliations:** 1 U.M.F. Doctoral School Craiova, University of Medicine and Pharmacy of Craiova, 200349 Craiova, Romania; 2 Leamna Pulmonology Hospital, 207129 Leamna, Romania; costin.streba@umfcv.ro; 3 Experimental Research Centre for Normal and Pathological Aging, University of Medicine and Pharmacy of Craiova, 200349 Craiova, Romania; 4 Department of Pulmonology, University of Medicine and Pharmacy of Craiova, 200349 Craiova, Romania

**Keywords:** COPD, pulmonary disease, depression, anxiety, antidepressants

## Abstract

**Background**: Chronic Obstructive Pulmonary Disease (COPD) is a prevalent, progressive condition often associated with psychiatric comorbidities such as depression and anxiety, which negatively affect disease progression, treatment adherence, and quality of life. **Methods**: A narrative review was conducted by searching PubMed and Google Scholar for English-language publications from January 2015 to July 2025. This type of review was selected to allow for a broad and integrative analysis of the current evidence on the association between COPD and psychiatric comorbidities, particularly depression and anxiety. To increase comprehensiveness, the reference lists of the included articles and systematic reviews were manually screened, and data extraction and evaluation were conducted independently by two reviewers. **Results**: Reported prevalence rates vary widely in COPD patients with depression ranging from 10 to 57% and anxiety from 7 to 50%, largely due to differences in patient populations, diagnostic tools, and disease severity. Identified risk factors include female gender, social isolation, smoking, low BMI, comorbidities, and systemic inflammation. These comorbidities are associated with increased exacerbations, higher hospitalization rates, and poorer clinical outcomes. While inhaled therapies may have limited impact on psychiatric symptoms, antidepressants must be used cautiously. Non-pharmacological interventions, including pulmonary rehabilitation, cognitive-behavioral therapy, physical activity, and social support, demonstrate clear benefits. **Conclusions**: Effective management of COPD requires integrated approaches that address both pulmonary and psychiatric components. Tailored interventions can improve clinical outcomes and quality of life. This review explores the bidirectional relationship between COPD and psychiatric disorders, aiming to highlight risk factors, diagnostic tools, and both pharmacological and non-pharmacological treatment strategies.

## 1. Introduction

Chronic Obstructive Pulmonary Disease (COPD) has been in recent years affecting around 10.6% of the global population with a rough estimate of around 480 million cases [1], becoming the third cause of death by 2030 worldwide [2]. Models predict that the global number of COPD cases among individuals aged 25 and older will increase by 23% between 2020 and 2050, reaching nearly 600 million patients worldwide by 2050 [1]. According to the Global Initiative for Chronic Obstructive Lung Disease (GOLD) 2023 classification, COPD is a heterogeneous respiratory condition, characterized by chronic symptoms such as shortness of breath, coughing, and expectoration caused by changes in the structures of the distal airways or in the alveoli (emphysematous changes) causing obstructive-like symptoms [3]. As a chronic and systemic disease, COPD will have an impact on other organs, contributing to the development of cardiovascular, neurological, and nutritional comorbidities [4]. Common COPD symptoms are also represented by fatigue and the inability to process their physical and some psychosocial needs [5]. These manifestations hold clinical significance for healthcare providers. A comprehensive assessment of symptom severity requires objective monitoring tools such as cough monitoring devices or home-based monitoring systems, which can facilitate timely interventions [6]. Nevertheless, challenges such as inadequate inhaler use [7] and treatment regimens that are either excessive or insufficient [8] often lead to suboptimal control and worsening of symptoms.

The incidence and prevalence of anxiety and depression are significantly higher in patients with COPD [9], with an estimation that one in three COPD patients presents depression associated with symptomatic manifestation [10]. The reported prevalence of anxiety among patients with COPD ranges from 7 to 50%, while the prevalence of depression varies between 10% and 57% [10]. The co-occurrence of COPD and depression is associated with a poorer patient outcome, increased mortality [11], and worsened prognosis [12] that might have an impact even on their caretakers, especially during hospitalization [13,14]. The incidence of comorbid COPD and depression in women has reduced lately, while the incidence among men has remained relatively stable. Nonetheless, both genders continue to exhibit increased in-hospital mortality rates within this patient population [15]. Alarmingly, fewer than half of COPD patients with comorbid depression receive mental health interventions during hospitalization, highlighting a critical gap in comprehensive patient care. This treatment gap is often attributed to factors such as underdiagnosis due to overlapping symptoms, stigma surrounding mental health, and the lack of integrated care pathways between respiratory and psychiatric services [16,17]. Moreover, patients with both COPD and depression exhibit a 1.9-fold increased suicide rate compared to those with COPD but no history of psychiatric disorders. This elevated risk may be explained by several overlapping factors: depressive symptoms are often underdiagnosed due to a symptom overlap with COPD; the chronic burden of breathlessness, physical disability, and social isolation can fuel hopelessness; shared inflammatory mechanisms may biologically link COPD with depression. Together, these factors contribute to a vicious cycle that worsens both psychological distress and pulmonary outcome [18,19,20,21]. This association disproportionately affects socioeconomically and socially vulnerable populations [22]. Patients diagnosed with depression tend to have a poor quality of life [23], even more so in association with COPD, with higher exacerbation rates, higher treatment costs, and poorer adherence to medical protocols, when compared to COPD patients with no comorbid psychiatric symptoms [24]. Patients with COPD also face an elevated risk of alcohol use problems [16], often also associated with depression and anxiety [25,26,27]. Despite this, individuals with post-traumatic stress disorder (PTSD) have a higher risk of COPD [28]. The management of loneliness of these patients is not enough [29], although palliative care can be an important tool in treating advanced COPD patients in managing their symptoms, but most of these patients will not access this type of care before death, highlighting a critical gap in end-of-life care [30].

Multiple biological and psychosocial mechanisms contribute to the association between COPD and depression. Shared pathways include systemic inflammation [31], immune system dysregulation [32], impaired diaphragmatic function, with hyperinflation and a shortening of the diaphragm muscle, rarely affecting the phrenic nerve [33]. Additional contributors are psychological conditions that increase the risk of COPD, even at low and moderate distress levels [34]. Furthermore, social isolation and poor interpersonal interactions, often affected by physical limitations, have been linked to a higher incidence of depression in COPD [35]. Although evidence supports the association, a specific biological biomarker that objectively validates this relationship through paraclinical means remains unidentified [36]. Recent systematic reviews, meta-analyses, and cross-sectional studies have identified protective factors such as increased physical activity, which has been shown to reduce hospitalization rates [37], and a higher sense of coherence may protect these patients [38]. However, certain additional risk factors include a low body mass index and heart dysrhythmias, increasing age, particularly middle and older adults [39,40], and the presence of chronic comorbidities and surgical procedures such as oophorectomies will increase the risk of a major depressive episode [29,41,42,43].

As a narrative review, this study aims to synthesize current evidence on the bidirectional relationship between COPD and psychiatric comorbidities, particularly depression and anxiety. The objective is to provide an integrative synthesis of the clinical implications of COPD–psychiatric comorbidity, to guide the development and application of targeted behavioral and therapeutic interventions, and to summarize the principal assessment methods employed for both COPD and these psychiatric disorders, thereby facilitating improved screening, monitoring, and management strategies in clinical practice.

## 2. Methods

We conducted an extensive literature search using databases such as PubMed and Google Scholar, prioritizing relevant articles on COPD, anxiety and depression, complete medical assessment of both pathologies, pharmacological and non-pharmacological treatment options, targeting English-language articles published between January 2015 and July 2025. The search terms employed mostly included “COPD” or “Chronic Obstructive Pulmonary Disease”, and “Depression” or “Anxiety”. To ensure complete coverage of the relevant literature, additional combined and derived terms were also employed, including “COPD/Depression Medication”, “COPD/Depression Non-Pharmacological Therapy”, and “COPD/Depression Assessment”.

Inclusion criteria were as follows:

- Studies focusing on patients with COPD.
- Articles addressing psychological comorbidities, specifically anxiety and/or depression.
- Studies reporting on medical assessments, pharmacological, or non-pharmacological interventions.
- Articles published in English within the specified time frame.
- Articles, systematic reviews, and meta-analyses providing original data or synthesized evidence relevant to COPD and mental health.

Exclusion criteria were as follows:

- Studies focusing on diseases other than COPD or not including COPD patients as a distinct population.
- Articles not addressing anxiety, depression, or mental health outcomes.
- Editorials, commentaries, or conference abstracts without sufficient data.
- Non-English publications.

In addition to the database searches, the reference lists of all eligible studies and related systematic reviews were manually screened to identify any additional studies. Data extraction and critical appraisal were performed independently by two reviewers. Each study was evaluated for study design, sample size, validity of measurements, and clarity of reported outcomes to ensure consistency and reliability in assessing study quality.

## 3. Risk Factors of Comorbid Depression and Anxiety in Patients with COPD

**Figure 1:**
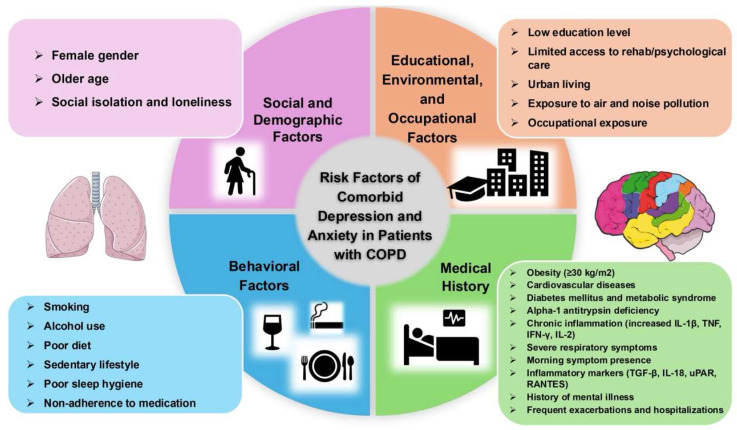
Diagram showing risk factors of comorbid depression and anxiety in patients with COPD.

Depression and anxiety frequently coexist with COPD, significantly complicating the clinical management and prognosis of affected patients. A wide range of risk factors, including social and demographic characteristics, educational and environmental influences, behavioral habits, and underlying medical history, contribute to the development and severity of these pathologies (Figure 1).

### 3.1. Social and Demographic Factors

Female COPD patients with psychiatric comorbidities may experience a greater negative impact on their subjective health outcomes compared to male patients [44]. The female gender represents an independent risk factor for developing psychiatric comorbidities in COPD, including depression and anxiety. Hormonal, psychosocial, and health behavior–related factors have been proposed as contributing mechanisms [45]. Further, women with COPD tend to report greater symptom burden, particularly in terms of dyspnea, and exhibit lower health-related quality of life compared to their male counterparts, despite similar levels of pulmonary impairment. This may be partially explained by anatomical differences proving that women have narrower airways, leading to greater mechanical limitations during breathing. These physiological factors, combined with heightened emotional responsiveness to somatic symptoms, may increase psychiatric vulnerability. Additionally, females are more likely to present with comorbid conditions such as heart failure, osteoporosis, and diabetes, which may further exacerbate psychological distress [45,46,47].

Older age increases vulnerability to both physical and mental health challenges. Older patients diagnosed with both COPD and depression demonstrate a heightened risk of disease advancement and reduced survival prospects [48]. On the other hand, recent studies suggest that younger individuals diagnosed with COPD exhibit a disproportionately higher risk of developing depressive symptoms. This increased vulnerability may be attributed to the earlier onset of disease, which often interferes with professional responsibilities, social functioning, and perceived quality of life [21].

Social isolation and loneliness are prevalent among individuals with COPD, primarily due to physical limitations and dependence on supplemental oxygen. These factors significantly increase the risk of psychological distress. Nearly one in six adults with COPD experience social isolation, and one in five report loneliness, with prevalence rates nearly twice as high in those requiring supplemental oxygen compared to the general population. Moreover, the incidence of depression is notably elevated among these individuals, affecting 36% of those experiencing depressive symptoms [49].

### 3.2. Educational, Environmental, and Occupational Factors

A low level of education, living in metropolitan areas, and occupational exposures [50] are important shared risk factors that contribute to both COPD progression and the development of depression and anxiety [51]. Individuals with lower education levels often lack health literacy, confidence in managing treatment, and self-management skills, which can exacerbate respiratory symptoms and increase vulnerability to mood disorders. Urban environments may further worsen outcomes due to higher air pollution, noise, and social isolation, all of which can aggravate pulmonary symptoms while promoting depressive and anxiety symptoms [50].

Similarly, occupational exposures, such as those in the textile or industrial sectors, can increase chronic respiratory inflammation and functional decline, while limited access to psychological support and rehabilitation programs hinders emotional adaptation [52], impeding long-term disease control and quality of life [53,54]. Collectively, these factors highlight how social, environmental, and occupational determinants converge to influence both the physiological progression of COPD and the neuropsychological pathways underlying depression and anxiety, emphasizing the importance of integrated management strategies.

### 3.3. Behavioral Factors

Behavioral factors also play a significant role in the association between COPD and depression, with smoking being a key contributor. Tobacco use not only exacerbates respiratory symptoms but also intensifies depressive manifestations [55,56]. In high-income countries, smoking is the primary cause of COPD, accounting for about 70% of cases [57]. Increased depressive symptoms are associated with a higher level of cigarette dependence among individuals with COPD [58]. Depression in COPD patients is linked to reduced survival rates and continued tobacco use. The severity of nicotine dependence and withdrawal symptoms experienced during smoking cessation efforts is often associated with psychiatric conditions such as anxiety and depression [59,60]. Moreover, smoking and alcohol use often reflect addiction-like behaviors, further complicating disease management and mental health outcomes [61].

Additionally, dietary patterns have been associated with better mental health outcomes. A cross-sectional study conducted in patients with COPD reported that certain dietary habits were linked to a lower prevalence of depressive symptoms, suggesting a potential role for nutrition in the mental health management of this population [62]. Research indicates that adherence to healthy diets, characterized by high intake of fruits, vegetables, whole grains, and omega-3 fatty acids, is linked to a reduction in depressive symptoms [62,63]. A positive correlation has been observed between mixed dietary patterns and improved pulmonary function, specifically reflected in higher FEV1 values. Following a healthy dietary pattern is associated with increased FEV1 and FVC measurements in both men and women. Notably, men adhering to healthier diets also exhibited higher FEV1/FVC ratios and a lower prevalence of COPD [62].

Inactive lifestyle will increase the risk of depression and anxiety, and would exacerbate COPD symptoms [51]. In contrast, patients with an active lifestyle that includes regular physical activity will have a better medical status [64].

Other behavioral factors, such as poor sleep hygiene and non-adherence to medication regimens, further compound the interplay between COPD and psychiatric comorbidities. Sleep disturbances are prevalent in individuals with COPD, with studies indicating that up to 70% of these patients experience poor sleep quality [65]. These disturbances, including insomnia and obstructive sleep apnea, are not only common but also exacerbate both pulmonary and psychological symptoms. Poor sleep quality is associated with increased fatigue, impaired emotional regulation, and heightened psychological distress [66,67,68]. Moreover, sleep problems are an established risk factor for developing depression and anxiety. They are among the most common residual symptoms in patients receiving treatment for depression, often leading to partial remission and an increased risk of relapse [69,70]. The bidirectional relationship between sleep disturbances and psychiatric disorders is well documented. Poor sleep can contribute to the onset and worsening of depression and anxiety, while these mental health conditions can further disrupt sleep patterns [71,72]. In COPD patients, the presence of sleep disturbances can initiate a vicious cycle. Respiratory symptoms such as dyspnea and nocturnal hypoxia can impair sleep quality, which in turn can exacerbate fatigue and emotional distress, leading to increased anxiety and depressive symptoms. This cycle complicates disease management and negatively impacts quality of life [73].

### 3.4. Medical History and Pathophysiological Mechanisms Involved

**Figure 2:**
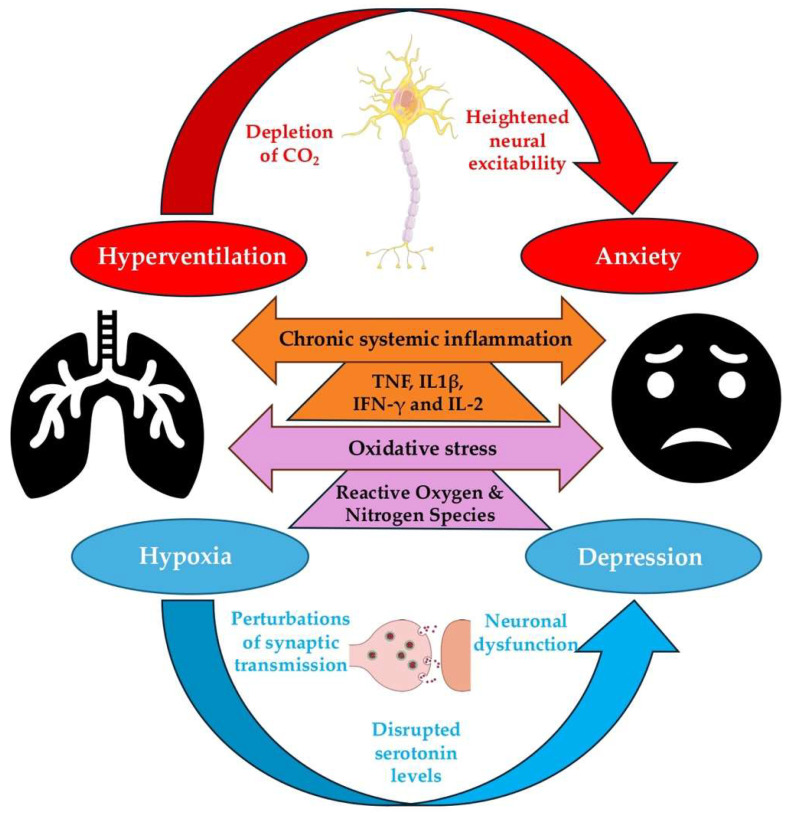
Shared pathophysiological mechanisms linking COPD with depression and anxiety.

A patient’s medical history plays a critical role in shaping the risk of comorbid depression in individuals with COPD, as pre-existing conditions and past illnesses influence both the onset and exacerbations of pathologies [74]. Notably, patients with obesity (≥30 kg/m^2^), with more than two associated comorbidities, have a higher depression rate [61]. Similarly, alpha 1 antitrypsin deficiency (AATD) is associated with more severe pulmonary impairment [75,76], and worsen symptoms such as cough, expectoration, and hemoptysis [77,78], all of which contribute to psychological distress. Emerging evidence highlights several pathophysiological mechanisms through which physiological dysregulation can contribute to the development of depression and anxiety. Hyperventilation, for instance, induces an anxiogenic effect through altered respiratory patterns that disrupt normal CO_2_ homeostasis, leading to heightened neural excitability and increased anxiety symptoms [79,80]. Concurrently, hypoxia, whether intermittent or chronic, can result in neuronal dysfunction, impairing synaptic plasticity and neurotransmitter balance, which in turn contributes to depressive symptomatology [81] (Figure 2).

Frequent exacerbations and recurrent hospitalizations further compound this burden, as they are associated with heightened anxiety, emotional instability, and feelings of helplessness [82,83]. In particular, anxiety tends to exert a more immediate effect on hospitalization risk during acute exacerbations [84], also increasing the cost rates [85]. The severity of respiratory manifestations, such as dyspnea, bronchiectasis, and the presence of multiple comorbidities, further increases susceptibility to anxiety, depression, and even secondary complications like osteoporosis, all of which negatively affect quality of life [86,87]. Additionally, the timing and pattern of symptom presentation may indicate disease severity; for instance, the presence of symptoms in the morning highlights a more severe pulmonary impairment, often associated with a lower FEV1 [88]. Patients with a FEV1 < 50% tend to experience a rapid decline in quality of life [89]. Elderly patients that have high Th1/Th1 levels and alterations in this ratio, or increased levels of Interleukin (IL)-18, transforming growth factor (TGF)-β, chemokine of regulated on activation, normal T cell expressed and secreted (RANTES), and urokinase plasminogen activator receptor (uPAR), are associated with a higher risk of depression development [31,48].

Cardiovascular diseases, such as heart failure, hypertension, and ischemic heart disease, are highly prevalent among patients with COPD and not only accelerate pulmonary decline but are also independently associated with a greater risk of depression and anxiety [90,91,92]. Similarly, metabolic disorders, including diabetes mellitus and metabolic syndrome, contribute to systemic inflammation and are recognized as predictors of poorer mental health outcomes [93,94]. Chronic systemic inflammation in COPD may also contribute to neuroinflammatory processes that underlie depression [95], and elevated levels of pro-inflammatory cytokines such as IL1β, IFN-γ, IL-2, and tumor necrosis factor (TNF) have been observed in patients suffering from both COPD and depression [96,97] (Figure 2).

## 4. Aggravating Factors

Aggravating factors are comparable to risk factors, given their shared role in influencing disease onset and progression. In the context of COPD and depression, these factors do not act in isolation but rather interact to amplify both physical and psychological symptoms. This bidirectional interplay underscores that aggravating factors serve as a bridge between the physiological deterioration characteristic of COPD and the neuropsychological mechanisms underlying mood disorders. One of the most frequently reported aggravating factors is an increase in exacerbations in female patients, which may reflect gender-related vulnerability to disease progression [98]. This factor can also be associated with exposure to air toxins such as tobacco and fuels [47].

Several behavioral factors, such as poor sleep quality, will impact physical daily activity, while smoking [99] and challenging life situations will increase depression-like symptoms in patients who present obstructive pulmonary disease [100]. Social isolation, poor family support system, financial problems, and occupations such as farming would worsen the patients’ symptoms, but higher income is associated with improved health status and access to care [101,102,103].

A complication that may result from these factors is sexual disorders in female patients, especially if both comorbidities are left untreated [104]. Undermining of dignity in severe cases of COPD is similar to neoplastic patients, as their wellbeing is affected [105], with high levels of death-related anxiety [106].

Physical factors like a low body mass index or obesity have been associated with a higher burden of comorbidities in patients with COPD and depression [107,108]. With regard to age-related factors, individuals under 55 years old had more frequent exacerbation symptoms compared to older patients, but comorbidities such as hypertension would worsen the depression if left untreated [109].

In elderly patients > 80 years old [110], unmanaged depression would lead to a higher risk of dementia in both genders [111], even though female patients over 75 years old would present with high use of antidepressant therapies and anxiolytic medications [112].

Paraclinical evaluation, such as reduced FEV1 and elevated CAT scores, has been identified as an aggravating factor [107], accompanied by laboratory findings of high inflammation factors such as IL1 beta, IFN-γ and IL-2, and TNF, would be present in patients with both COPD and depression [96,97].

In addition, treatment-related factors may worsen the patients’ status, as treatment with inhaled corticosteroids may have a higher rate of admission in a psychiatric ward and an increase in administration of antidepressant medication, raising concerns about the neuropsychiatric side effects of certain COPD pharmacotherapies in the vulnerable patient population [113]. Overall, several aggravating factors identified are modifiable, such as smoking, poor sleep quality, physical inactivity, social isolation, and inadequate treatment adherence, and can be targeted through behavioral and clinical interventions. In contrast, non-modifiable factors such as age, gender, and existing comorbidities require tailored management strategies to mitigate their impact.

## 5. Evaluation of COPD and Comorbid Depression or Anxiety

Given the negative impact of depressive and anxiety symptoms on COPD outcomes, a variety of validated clinical tools are necessary to adequately evaluate both pulmonary function and mental health status in affected individuals.

### 5.1. Pulmonary COPD Evaluation

The clinical evaluation of COPD (Table 1) is not limited to respiratory impairment but is closely intertwined with the identification of psychiatric comorbidities, particularly depression and anxiety. Several standardized measures used to assess COPD severity also provide indirect insight into the psychological burden of the disease [114,115].

This relationship becomes particularly evident when examining functional- and symptom-based assessments, which not only quantify disease severity, but also capture its psychosocial consequences. To assess functional scores, the COPD Assessment Test (CAT) or St. George’s Respiratory Questionnaire (SGRQ) can be used, which, in the case of patients with depression, has been observed to increase the values of the test [116,117]. For dyspnea assessment, the Modified Medical Research Council Dyspnea Scale (mMRC) can be applied, which, in the case of patients with dyspnea, determines an increase in dyspneic symptoms [118]. Higher scores on the 6 min walk test, mMRC dyspnea scale, and CAT scores indicate worse functional status and symptom burden, and have been consistently associated with increased levels of depressive and anxiety symptoms. For instance, patients with severe COPD demonstrate significantly higher anxiety and depression scores, as well as reduced self-compassion and physical functioning, compared to those with moderate disease severity. These findings support the role of COPD severity as both a clinical and psychological determinant, highlighting the need for mental health screening in patients with advanced disease stages [119].

Spirometry remains the gold standard for assessing lung function in patients with COPD. It is a non-invasive, safe, and widely accessible method that provides key diagnostic parameters, including the FEV_1_ and the FEV_1_/FVC ratio, both of which are essential for confirming airflow limitation [120]. In some studies, it has been observed that depression has a negative effect on long-term function [121]. As an addition to spirometry, we can also add the transfer factor for carbon monoxide (TLCO), which can be used to measure the amount of carbon monoxide and the transfer constant through the alveolar–capillary membrane [122]. Furthermore, body plethysmography for functional residual capacity (FRCpleth) and specific airway resistance (sRAW), as well as residual volume (RV), total lung capacity (TLC), and inspiratory capacity (IC) parameters that are particularly used in cases involving hyperinflation or air trapping [123,124].

Depression has also been associated with lower physical performance scores on the BODE index [125], a well-known predictor of the risk of death from any cause and from respiratory causes among patients with COPD [126].

Radiological evaluation also contributes to functional assessment. The pulmonary emphysema index, determined through high-resolution computed tomography (HRCT), has shown a positive correlation with the FEV_1_/FVC ratio and can be a valuable tool for identifying early structural changes in individuals at risk of COPD [127].

Moreover, AATD is associated with panacinar emphysema, which is highest in COPD patients [128]. Individuals with COPD show changes in biological values regarding inflammatory markers such as C-reactive protein (CRP), fibrinogen, TNF-α, IL-6, and IL-8, which in most cases are increased [129].

### 5.2. Depression and Anxiety Evaluation

Several validated psychological assessment tools are employed to evaluate depression and anxiety in patients with COPD (Table 2). Commonly used instruments include Hospital Anxiety and Depression Scale-Anxiety (HADS-A), Hospital Anxiety and Depression Scale-Depression (HADS-D), Beck Depression Inventory II (BDI), and Beck Anxiety Inventory (BAI), which are useful for quantifying depression and anxiety, presenting a series of questions related to the patients’ mood, how they feel, if they wake up at night or if they manage to rest, feelings of fear, insecurity, or pressure [130,131].

In a more personalized intervention, Tailored intervention for ANxiety and DEpression Management (TANDEM) was applied using the previously mentioned questionnaires [132], proving a suitable approach combining psychological tools for better COPD-specific mental health management.

In addition to general mental health instruments, disease-specific tools like the COPD-Anxiety Questionnaire (CAF), which assesses dyspnea, physical activity, social exclusion, or disease progression, offers a more COPD-focused psychological evaluation [133].

Alongside standard tools such as HADS and BDI, several other self-report questionnaires have been utilized to assess psychological comorbidities in patients with COPD. These methods included scores on the Self-Rating Anxiety Scale (SAS) and Self-Rating Depression Scale (SDS) [134] or the depression scale (DEPS) questionnaire [135].

In patients with chronic respiratory failure, the Edmonton Symptom Assessment Scale (ESAS) was used to better assess depressive symptoms [135] in the case of episodes of severe depression, but also in episodes of anxiety, and DSM-IV (Diagnostic and Statistical Manual) was applied for a better definition of them [136].

Importantly, patients with COPD and coexisting depression or anxiety have lower lung function results compared to those without psychological comorbidities. These findings suggest a bidirectional relationship between mental health status and physiological disease burden in COPD [137].

## 6. Therapeutic Strategies

Depression and anxiety can alter therapeutic decision-making in COPD by affecting patients’ adherence, perception of symptoms, and overall engagement with treatment. The presence of psychiatric comorbidities may complicate both pharmacological and non-pharmacological interventions, sometimes requiring adjustments in management strategies. Conversely, treatments for depression and anxiety, whether pharmacological or behavioral, can also influence COPD outcomes, either positively, by improving adherence and quality of life, or negatively, through side effects or interactions with respiratory medications. These complex interactions highlight the need for an integrated, multidisciplinary approach to treatment that simultaneously addresses both respiratory and psychological health [138,139,140].

### 6.1. Pharmacological Treatment

Pharmacological treatment plays a critical role in terms of decreasing airway inflammation, dilating the airways, and automatically reducing exacerbations [141]. COPD management is guided by the Global Initiative for Chronic Obstructive Lung Disease (GOLD), which provides regularly updated and evidence-based recommendations aimed at optimizing the diagnosis, treatment, and long-term care of patients with COPD [142].

Compliance of patients with COPD treatment is influenced by the presence of depression and anxiety. Sometimes, patients with a recent depression diagnosis would have a lower adherence to inhalation treatment [143]. Excessive use of inhaled corticosteroid (ICS) therapy, defined as >125% of expected utilization based on the prescribed dosage regimen, theoretical exposure duration, and medication supply, has been associated with the development of psychological dependence. Patients in this category often report feeling unwell or anxious when their inhalation device is unavailable or when medication doses are restricted [144]. On the other hand, anxious patients frequently present with somatic symptoms such as shortness of breath or chest tightness, even in the absence of any underlying respiratory condition like COPD. These manifestations, often driven by psychological distress, can lead to unnecessary use of inhaled corticosteroids as patients seek relief from symptoms that are not rooted in a physiological pathology [145,146].

Conversely, underuse of ICS therapy, defined as <50% of expected utilization using the same criteria, is frequently driven by patients’ perception of clinical stability or a lack of perceived need for treatment, leading them to intentionally reduce [144]. Moreover, long-acting beta2 agonist and long-acting muscarinic antagonist had no significant impact on HADS scores in patients with newly diagnosed COPD, but bronchodilator therapy proved ineffective on depression and anxiety [147,148]. On the other hand, patients with corticosteroid inhalation medication proved to be more susceptible to depression, compared to patients with non-steroid treatment [149].

In some situations, persistent dyspnea can be treated with opioids, but it is important to manage it through non-pharmacological methods. However, there is reluctance to prescribe opioids to manage dyspnea in COPD [150]. Concerningly, COPD and depression have been associated with long-term opioid use and substance abuse [151].

An alternative to consider is revefenacin, a long-acting muscarinic antagonist used in the management of COPD, especially since the safety profile of revefenacin was not affected by comorbid anxiety or depression [152]. However, dyspnea therapies such as dronabinol, a cannabinoid agent used experimentally for dyspnea relief, showed no improvement compared to placebo [153].

**Figure 3:**
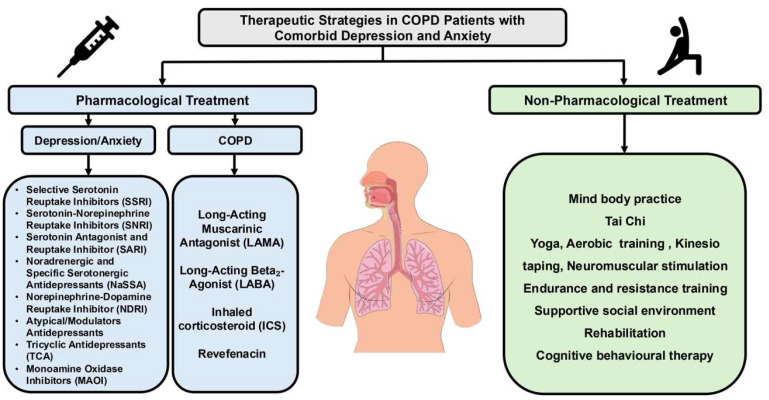
Diagram showing pharmacological and non-pharmacological therapeutic strategies in COPD patients with comorbid depression and anxiety.

Antidepressant treatment is complex, and its use and dosage depend on the individual patient, their symptoms, and the specific neurotransmitters the medication targets (Figure 3) [154].

Appropriate pharmacological management of depression can increase the use of and adherence to maintenance medication required for COPD. In addition, antidepressant treatment may be a major factor in improving medication adherence in COPD patients who also have depressive symptoms [155,156]. Furthermore, it was highlighted that depressive symptoms improved more in the groups that received medication compared to those who received only a placebo or educational intervention [157].

While antidepressants such as SSRIs and SNRIs are key to treating depression, studies have raised concerns about their safety in COPD patients [158]. Both SNRIs and SSRIs have been linked to an increased risk of pneumonia in elderly COPD patients, according to observational and retrospective population-based cohort studies. While these findings raise safety concerns, especially regarding respiratory-related morbidity and mortality, causal relationships have not been definitively established [159]. However, methodological limitations of the study suggest these findings should be interpreted with caution [158]. Furthermore, although SSRI and Mirtazapine proved not to ameliorate shortness of breath or spirometry evaluation, but can have a benefit in functional tests such as the 6 min walking test [160,161]. Insomnia is frequently observed alongside comorbid conditions commonly associated with COPD, including depression and anxiety, and it has been linked with an increased risk of COPD exacerbations [162,163]. Mirtazapine and trazodone are frequently utilized in this clinical context. However, evidence supporting their long-term efficacy remains limited [163]. Bupropion, a dopaminergic agent, is often prescribed for smoking cessation, yet concerns exist regarding its potential to suppress ventilatory responses to hypoxemia and hypercapnia through dopamine-mediated inhibition of carotid body chemoreception [158,164]. Another potential approach involves the use of selective orexin receptor antagonists (SORA1s), a novel strategy in the treatment of anxiety disorders. Unlike benzodiazepines, SORA1s do not exhibit the pronounced sedative effects typically associated with traditional anxiolytics [165].

Emerging evidence suggests that certain antidepressants may provide dual benefits in patients with COPD and comorbid depression or anxiety. Nortriptyline, a tricyclic antidepressant, has shown promising results in a randomized controlled trial, where it significantly improved not only depressive and anxiety symptoms but also respiratory function and overall physical performance in COPD patients. In a randomized trial, it was proven that nortriptyline, a tricyclic antidepressant, not only improved depressive and anxiety symptoms, but also respiratory symptoms and daily functioning in COPD patients [24].

Additionally, when a comparison was made between a control group and an intervention group, the latter significantly reduced the incidence of readmissions. The intervention group showed a trend towards a significant decrease in depression as measured by the HADS, but no decrease in anxiety as measured by the HADS. However, significant improvements in CAT scores were observed for participants in the intervention group. Also, minor differences were found in the use of inhalation therapy [166].

### 6.2. Non-Pharmacological Treatment

Non-pharmacological interventions represent a crucial component in the comprehensive management of patients with COPD and psychiatric comorbidities. Daily physical therapies such as mind–body practice, yoga, aerobic exercise, Tai Chi, performed within an individual’s physical capacity, yield a better outcome in the symptoms of psychiatric diseases, and even in COPD management. These interventions are supported by evidence from observational studies, as well as randomized controlled trials and double-blind, placebo-controlled trials, indicating a growing body of research that supports their effectiveness in managing both psychiatric symptoms and COPD-related outcomes [167,168,169,170].

Also, other supportive therapies such as dignity therapy, kinesio taping [171], neuromuscular electrical stimulation [172], even endurance and resistance training [173], have demonstrated positive outcomes on depressive and even pulmonary symptoms.

Rehabilitation is a well-established intervention for patients with COPD, encompassing structured physical exercise, patient education, nutritional advice, and psychosocial support. Beyond improving exercise tolerance and lung function, rehabilitation has been shown to positively affect mental health outcomes, including symptoms of depression and anxiety. By targeting both physical and psychological domains, rehabilitation represents an integrated approach that may slow disease progression, reduce symptom burden, and improve overall quality of life in patients with COPD and comorbid depression or anxiety [174]. On the other hand, pulmonary tele-rehabilitation was not superior to classic pulmonary rehab [175,176], though in some cases, digital physical rehabilitation was proven somewhat more effective in COPD patients, but showed no improvement in social functioning aspects for these patients [177].

In addition to structured physical exercise, pulmonary rehabilitation programs often include psychosocial education, which plays a crucial role in managing both COPD and associated mood disorders. Educational interventions can improve treatment adherence, self-management skills, and coping strategies, thereby positively influencing disease progression and symptom control. Furthermore, psychosocial education addresses anxiety and depressive symptoms by providing patients with strategies to manage stress, improve resilience, and enhance social support networks [178].

Patients who received individualized nursing as part of a therapeutic ward environment, provided with psychological counseling and health education, managed respiratory and systemic complications with diet, oxygen, and symptom-focused care, promoted rehabilitation exercises, receiving individualized home-based nursing support after discharge, and engaging patients in educational sessions to enhance disease understanding and self-management, performed better in the 6 minute walk test and showed a reduction in hospitalization rates [179].

Regarding social support, COPD patients who received higher social support, family assistance during activities, and even during inpatient care, had superior outcomes regarding their respiratory manifestations and functional outcomes [180,181,182].

Importantly, a supportive social environment will ameliorate the anxiety and depressive symptoms [183], even though this support may be given online [184]. Additionally, telemedicine reexamination after discharge can be more cost-efficient and as good as in-person follow-up [185]. Conversely, automated oxygen administration, despite optimal oxygen titration, does not improve COPD or psychological symptoms [186].

Another tool is represented by cognitive-behavioral therapy, which would reduce anxiety and depression and improve quality of life [187]. A nurse-coordinated behavioral therapy would even lead to lower HADS-A scores and lower hospitalization rates [188], but it must be highlighted that this type of therapy works best when integrated into pulmonary rehab for at least eight weeks [189].

## 7. Future Directions

### 7.1. Clinical Integration and Risk Stratification

There is a clear need for more prospective studies [190], that will address the integration of mental health and focus on the recommendation regarding mental and physical management of COPD patients, then these could help provide the best patient care [191]. One risk category of COPD patients may be smokers, who, one in five, have unmedicated symptoms that will need a multidimensional approach incorporating both physical and psychological components for optimized care [192].

### 7.2. Behavioral Interventions

Better management of mental symptoms has been shown to reduce pulmonary exacerbation, improving their overall status [193]. In parallel, promoting daily physical activity not only benefits lung function but also exerts positive effects on mental wellbeing, particularly when integrated into socially responsive frameworks aimed at addressing inequality, unhealthy behavior, and mood disorders. Integrating daily physical activities may improve mental health [168,194], but a focus on nutrition, especially muscle mass and obesity awareness, would have an impact on COPD [195]. Offering lung function testing to individuals who smoke may serve as an effective intervention to support smoking cessation by highlighting the negative impact of tobacco use on respiratory health [196].

### 7.3. Experimental and Translational Research

Recent murine models may rise new treatment possibilities such as the following: phycocyanin (PC)—a pigment–protein complex found in cyanobacteria—exerts anti-inflammatory and antioxidant effects in experimental models of COPD by regulating heme oxygenase-1 (HO-1) and nicotinamide adenine dinucleotide phosphate (NADPH) dehydrogenase quinone 1 (NQO1) in lung tissue, and by reducing NADPH oxidase 2 (NOX2) expression in pulmonary macrophages, it helped alleviate oxidative stress and inflammation [197]. Preclinical murine models suggest that mesenchymal stem cell (MSC) therapy may have protective effects in COPD by modulating the immune response, decreasing inflammation, and improving bacterial clearance. These findings highlight the potential of MSCs as a novel adjunct treatment, particularly for COPD patients with frequent bacterial exacerbations [198]. On the other hand, cigarette smoke-induced lung inflammation leads to anxiety-like behavior and social recognition deficits in murine models, affecting the central nervous system. Interventions such as NOX2 inhibition (e.g., apocynin) show promise in mitigating both pulmonary and neurobehavioral consequences of smoke exposure [199]. Translational murine studies are warranted to assess whether targeted nutritional interventions can modulate neuropsychiatric comorbidities in COPD. Specifically, supplementation with omega-3, tryptophan, vitamin D, and prebiotics may improve anxiety, cognition, and neuroinflammation. Future clinical trials should evaluate cognitive-behavioral, immunological, and quality-of-life outcomes [200].

### 7.4. Multimodal and Personalized Interventions

Recent evidence indicates that combining interventions integrating physical activity (e.g., pulmonary rehabilitation or physical activity behavioral modification [BPA]) with psychological support, such as cognitive-behavioral therapy (CBT), yields superior outcomes in patients with COPD and comorbid anxiety or depression. Unlike isolated psychological or rehabilitation interventions, these multimodal programs have demonstrated greater improvements in daily physical activity levels, exercise capacity, anxiety symptoms (notably ~2-point reduction in HADS-A), and overall quality-of-life outcomes, compared to CBT combined with rehabilitation alone [201].

Future strategies should prioritize personalized treatment plans, taking into account the individual clinical, social, and psychological profiles of each patient [202]. Moreover, an intervention as early as possible [203] and with interdisciplinary boards [204] will have a positive impact on the care of COPD patients.

## 8. Conclusions

COPD represents a multifaceted condition in which psychiatric comorbidities, particularly depression and anxiety, exert a profound influence on disease progression, therapeutic adherence, healthcare integration, and patient-reported outcomes. While prior reviews have addressed the epidemiological burden of these comorbidities, the present narrative review extends the existing body of literature by offering an integrated synthesis of current evidence regarding diagnostic methodologies, risk stratification (including both modifiable and non-modifiable factors), and a range of therapeutic interventions, encompassing both pharmacological and non-pharmacological domains.

Notably, this review underscores emerging areas of interest, such as the neuropsychiatric impact of inhaled pharmacotherapies, the utility of lifestyle-based interventions (e.g., mind–body practices and nutritional modulation), and findings from translational research, including murine models and stem cell therapies. The review advocates for a paradigm shift towards personalized management strategies, wherein clinical, psychological, and social dimensions are concurrently addressed.

For clinicians, key implications include the necessity of systematic mental health screening in COPD populations, awareness of the bidirectional interplay between psychiatric and pulmonary pathology, and the implementation of interdisciplinary care models that integrate behavioral, psychosocial, and rehabilitative modalities. Future research should prioritize longitudinal and interventional studies to validate these approaches and support the development of evidence-based, patient-centered care pathways for individuals with COPD and comorbid mental health disorders.

## Figures and Tables

**Table 1:** Evaluation instruments for pulmonary function in COPD Patients.

	Method/Scale	Type of Measurement	Description
**Pulmonary function**	Spirometry	Non-invasive test of airflow limitation	Gold standard for diagnosing COPD and measuring lung function severity (FEV_1_, FEV_1_/FVC ratio)
	Transfer factor for carbon monoxide (TLCO)	Gas diffusion test	Assesses gas exchange efficiency across the alveolar–capillary membrane.
	Body plethysmography	Lung volume and airway resistance measurement	Allows to assess functional residual capacity (FRCpleth) and specific airway resistances (sRAW), as well as residual volume (RV), total lung capacity (TLC), and inspiratory capacity (IC) parameters.
	COPD Assessment Test (CAT)	Self-administered questionnaire	Measures health-related quality of life and evaluates symptom burden; scores often increase in patients with depression.
	St. George’s Respiratory Questionnaire (SGRQ)	Self-administered questionnaire	Designed to measure health impairment in patients with asthma and COPD.
	Modified Medical Research Council Dyspnea Scale (mMRC)	Scale from 0 to 4	Used to assess the degree of baseline functional disability due to dyspnea.
	Body mass index, airflow Obstruction, Dyspnea, and Exercise capacity (BODE) index	Simple multidimensional grading system	Predicts the risk of death from any cause and from respiratory causes among patients with COPD.
	Pulmonary emphysema index, determined through high-resolution computed tomography (HRCT)	Imaging (High-Resolution CT)	Detects structural changes and correlates with spirometric values.
	Biomarkers (CRP, IL-6, IL-8, TNF-α, fibrinogen)	Blood analysis	Elevated in COPD and associated with systemic inflammation and comorbid depression.
	Alpha-1 Antitrypsin Deficiency Testing (AATD)	Genetic/serological testing	Identifies risk of panacinar emphysema, often associated with severe COPD forms.

**Table 2:** Evaluation instruments for comorbid depression/anxiety in COPD Patients.

	Method/Scale	Type of Measurement	Description
**Depression and Anxiety**	Hospital Anxiety and Depression Scale-Depression (HADS-D)	Self-assessment scale	Used for quantifying depression and anxiety symptoms, presents a series of questions related to the patients’ mood, how they feel, if they wake up at night or if they manage to rest, feelings of fear, insecurity or pressure.
	Hospital Anxiety and Depression Scale-Anxiety (HADS-A)		
	Beck Depression Inventory II (BDI)		
	Beck Anxiety Inventory (BAI)		
	Tailored intervention for ANxiety and DEpression Management (TANDEM)	Multi-instrument personalized intervention	Tailored approach combining psychological tools for better COPD-specific mental health management.
	COPD-Anxiety Questionnaire (CAF)	COPD-specific psychometric tool	Evaluates anxiety related to dyspnea, physical activity, social exclusion, or disease progression.
	Self-Rating Anxiety Scale (SAS)	Self-administered questionnaire	Assesses depression and anxiety severity based on self-reported symptoms.
	Self-Rating Depression Scale (SDS)		
	Depression scale (DEPS) questionnaire		Identifies depressive symptoms, especially in chronic disease contexts.
	Edmonton Symptom Assessment Scale (ESAS)	Scale from 0 (no symptoms) to 10 (worst possible symptom)	Used in patients with chronic respiratory failure to assess depression/anxiety burden.
	Diagnostic and Statistical Manual (DSM-IV)	Standardized classification system	Gold standard for diagnosing clinical depression or anxiety disorders, based on specific criteria related to symptoms, duration, and functional impact.

## Abbreviations

The following abbreviations are used in this manuscript:

AATD	Another key factor is alpha-1 antitrypsin deficiency
BAI	Beck Anxiety Inventory
BDI	Beck Depression Inventory II
BODE	Body mass index, airflow Obstruction, Dyspnea, and Exercise capacity
BPA	Activity behavioral modification
CAF	COPD-Anxiety Questionnaire
CAT	COPD Assessment Test
CBT	Cognitive-behavioral therapy
COPD	Chronic Obstructive Pulmonary Disease
CRP	C-reactive protein
DEPS	Depression scale questionnaire
DSM-IV	Diagnostic and Statistical Manual
ESAS	Edmonton Symptom Assessment Scale
GOLD	Global Initiative for Chronic Obstructive Lung Disease
FEV1	Forced expiratory volume in one second
FEV1/FVC	Tiffeneau index
FRCpleth	Body plethysmography for functional residual capacity
FVC	Forced vital capacity
HADS-A	Hospital Anxiety and Depression Scale-Anxiety
HADS-D	Hospital Anxiety and Depression Scale-Depression
HAMA	Hamilton Anxiety Rating Scale
HAMD	Hamilton Depression Rating Scale
HO-1	Heme oxygenase-1
HRCT	High-resolution computed tomography
IC	Inspiratory capacity
ICS	Inhaled corticosteroid
IL	Interleukin
LABA	Long-acting beta2 agonist
LAMA	Long-acting muscarinic antagonist
MAOI	Monoamine oxidase inhibitors
MEP	Expiratory pressures
MIP	Maximum inspiratory
mMRC	Modified Medical Research Council Dyspnea Scale
MSC	Mesenchymal stem cell
NAD(P)H	Nicotinamide adenine dinucleotide phosphate
NaSSAs	Noradrenergic and specific serotonergic antidepressants
NDRI	Norepinephrine-dopamine reuptake inhibitor
NOX2	Nicotinamide adenine dinucleotide phosphate oxidase 2
NQO1	Nicotinamide adenine dinucleotide phosphate dehydrogenase quinone 1
PC	Phycocyanin
PRISMA	Preferred Reporting Items for Systematic Reviews and Meta-Analyses
PTSD	Post-traumatic stress disorder
RANTES	Regulated on activation, normal T cell expressed and secreted
RV	Residual volume
SARI	Serotonin antagonist and reuptake inhibitor
SAS	Self-Rating Anxiety Scale
SDS	Self-Rating Depression Scale
SGRQ	St. George’s Respiratory Questionnaire
SNRIs	Serotonin–norepinephrine reuptake inhibitors
SORA1s	Selective orexin receptor antagonists
sRAW	Airway resistances
SSRIs	Selective serotonin reuptake inhibitors
TANDEM	Tailored intervention for ANxiety and DEpression Management
TCAs	Tricyclic antidepressants
TGF	Transforming growth factor
TLC	Total lung capacity
TLCO	Transfer factor for carbon monoxide
TNF	Tumor necrosis factor
UPAR	Urokinase plasminogen activator receptor
